# Impact of environmental air pollution on respiratory health and function

**DOI:** 10.14814/phy2.70006

**Published:** 2024-08-22

**Authors:** Samuel Wallbanks, Benjamin Griffiths, Maximillian Thomas, Oliver J. Price, Karl P. Sylvester

**Affiliations:** ^1^ Birmingham Heartlands Hospital University Hospitals Birmingham Birmingham UK; ^2^ School of Biomedical Sciences, Faculty of Biological Sciences University of Leeds Leeds UK; ^3^ Respiratory Physiology University Hospitals Sussex NHS Foundation Trust Brighton UK; ^4^ Department of Respiratory Medicine Leeds Teaching Hospitals NHS Trust Leeds UK; ^5^ Respiratory Physiology Papworth Hospital NHS Foundation Trust Cambridge UK; ^6^ Respiratory Physiology Cambridge University Hospitals NHS Foundation Trust Cambridge UK

**Keywords:** airway, health inequalities, pollution, respiratory physiology

## Abstract

Environmental air pollution presents a considerable risk to global respiratory health. If critical levels are exceeded, inhaled pollutants can lead to the development of respiratory dysfunction and provoke exacerbation in those with pre‐existing chronic respiratory disease. Over 90% of the global population currently reside in areas where environmental air pollution is considered excessive—with adverse effects ranging from acute airway irritation to complex immunomodulatory alterations. This narrative review provides an up‐to‐date perspective concerning the impact of environmental air pollution on respiratory health and function and describes the underpinning mechanisms that contribute to the development and progression of chronic respiratory disease.

## 1 INTRODUCTION

Environmental air pollution refers to unwanted chemical, physical, or biological material in the atmosphere that can be detrimental to human health (Landrigan, 2017). Despite clear recommendations from the World Health Organization (WHO) concerning maximum annual and seasonal levels of pollutants (WHO, n.d.), >90% of the global population currently reside in industrialized cities where environmental air contains excessive particulate matter (PM), ozone, and oxides of sulfur and nitrogen. Environmental air pollution is now recognized as a leading cause of morbidity and mortality with an estimated 4.2 million annual deaths (WHO, n.d.). The respiratory system is particularly vulnerable to the effects of air pollution, with recent epidemiological studies indicating an increased incidence of acute respiratory infection and heightened risk of developing lung cancer, asthma, and chronic obstructive pulmonary disease (COPD) (Gourd, 2022; Guo et al., 2018).

The deleterious impact of air pollution on respiratory health can be quantified by evaluating lung function (Agustí et al., 2017) – which incorporates objective measurement of airway caliber, resistance, conductance, lung volumes, and alveolar‐capillary gas exchange (Sylvester et al., 2020). Collectively, these physiological parameters may be impacted by acute and chronic exposures to specific pollutants. The most common pollutants that have been studied in recent years include PM, ozone, and nitrogen dioxide (NO_2_) (WHO, n.d.), which are ubiquitous within the environmental air, but are not always present at concentrations that cause an increased risk of respiratory illness or dysfunction.

The impact of environmental air pollution is not distributed proportionally within populations, with variable effects dependent on cumulative exposure and individual susceptibility. For example, children, older adults, and those with pre‐existing respiratory conditions, and/or living in relative or absolute deprivation appear to be at the greatest risk of the effects of air pollution (Aithal et al., 2023; Marmot & Bell, 2018). Despite this, there remains a paucity of evidence evaluating the effects of environmental air pollutants on respiratory function in these groups.

The purpose of this narrative review is to provide an up‐to‐date perspective concerning the impact of environmental air pollution on respiratory health and function, and describe the underpinning mechanisms that contribute to development and progression of chronic respiratory disease. In accordance with the Scale for the Assessment of Narrative Review Articles (SANRA) (Baethge et al., 2019), peer‐reviewed scientific literature published between 2018 and 2024 were identified in PubMed and Google Scholar using broad search terms such as “lung function” or “pulmonary function” and “air pollution” or “environmental pollution” or “ambient pollution.”

## 2 EPIDEMIOLOGY OF ENVIRONMENTAL AIR POLLUTION

Global air quality guidance is primarily based on complex modeling of the mass concentration of atmospheric air pollutants and the associated health impact of exposure (Lelieveld et al., 2015). This approach is limited, however, as pollutants originating from different sources often have different toxicity, composition, and chemical structures (Li et al., 2019). Indeed, recent studies indicate greater adverse health effects and mortality with each unit increase in fine particulate matter (PM_2.5_) in Eastern Chinese Cities (e.g., Shanghai, Hangzhou) when compared to the rest of China. This is despite broadly similar characteristics and environmental conditions in these populations. In Europe and the Unites States, greater mortality occurs for a given change in PM_2.5_ relative to China (Chen et al., 2017). Whilst increased cardiovascular health risk in Western populations is a potential explanatory factor, there is growing evidence to suggest unequal toxicities of pollution across the globe (Li et al., 2019).

Environmental air often contains a mixture of polluting and non‐polluting PM from natural and anthropogenic processes. The most common natural sources of environmental pollution include sand and dust storms, volcanic activity, and forest fires (Li et al., 2019). Anthropogenic sources include fuel combustion, agriculture, waste incineration, and traffic‐related air pollution. Fuel combustion remains the largest source of environmental air pollution globally, with 85% of all environmental PM_2.5_ and almost all oxidizing gases generated through this source (OECD I, 2016). Air pollution levels differ regionally according to factors such as economic practices, variations in climate and predominant fuel sources (Lelieveld et al., 2015).

Despite increasing awareness concerning the health impact of outdoor air quality, mortality rates have risen by 66% over the past two decades (Fuller et al., 2022). Indeed, recent statistics from the 2019 Lancet Commission on Planetary Health indicate that over 4‐million deaths were attributable to PM_2.5_ and 370,000 related to environmental ozone exposure. High environmental air pollution is associated with increased incidence of respiratory tract infections (Asri et al., 2021), COPD (Guo et al., 2018) and lung cancers (Gourd, 2022). The prevalence of asthma is also higher in polluted areas (Orellano et al., 2017). Additionally, acute spikes in air pollution correlate with respiratory symptoms and exacerbation in people with chronic respiratory disease—which ultimately leads to increased healthcare utilization (Hunt et al., 2003; Johannson et al., 2014).

The effects of environmental air pollution on respiratory health are not distributed equally around the world. Approximately three quarters of the population live in low and middle‐income countries, where 90% of the air‐pollution related mortalities occur (Fuller et al., 2022). Despite evidence to support the health benefits of reducing air pollution (Gauderman et al., 2015), this remains a challenge for low‐ and middle‐income countries due to the heavy economic reliance on highly polluting processes (Moser & Satterthwaite, 2010). The effects of climate change and extreme weather also have a significant impact in these countries (Keswani et al., 2022). A shared global responsibility is therefore required to mitigate the disproportionate health impact in highly polluted areas.

## 3 PATHOPHYSIOLOGICAL MECHANISMS OF LUNG FUNCTION IMPAIRMENT

Environmental air pollutants implicated in the development and progression of lung function impairment include PM, oxides of nitrogen (NO_x_), sulfur dioxide (SO_2_), ozone (O_3_), and volatile organic compounds. Of these, each compound has a specific mechanism of action with variable effects depending on the concentration, deposition, and substance toxicity (Mossman et al., 2007).

### 3.1 Factors impacting inhaled particle deposition

The respiratory system is particularly vulnerable to the effects of air pollution given its large surface area and role as an interface between the circulation and environmental air (Cohen et al., 2017). The properties of the inhaled pollutant modulate the deposition, retention, and overall effect on the respiratory system (see Table 1 for summary of pollutant characteristics (Weill, 2020)). For instance, the location of pollutant deposition modulates the type of symptoms observed: inflammation of the upper respiratory tract leads to rhinitis, pharyngitis, and laryngitis; bronchial inflammation leads to bronchitis and bronchopneumonia; and parenchymal inflammation leads to pulmonary oedema and pneumonia (Weill, 2020).

**TABLE 1:** Overview of common air pollutants that potentially contribute to the development of respiratory disease.

	Pollutant properties					
	Size	Solubility	Coagulability/reactivity	Density	Deposition	Respiratory disease
PM_10_	↑	‐^b^	↓	↔^c^	Nasopharyngeal	Asthma (Choi et al., 2020; Schultz et al., 2016); rhinitis (Lin et al., 2021); COPD (Doiron et al., 2019; Zemp et al., 1999); URTIs (Ziou et al., 2022).
PM_2.5_	↓	‐^b^	↑	↔^c^	Bronchioalveolar	Asthma exacerbation (Habre et al., 2014); rhinitis (Lin et al., 2021; Montgomery et al., 2020); COPD (Doiron et al., 2019; Schikowski et al., 2014); LRTIs (Asri et al., 2021); Lung cancer (Huang et al., 2017); ILD (Harari et al., 2020).
PM_0.1_	↓↓	‐^b^	↑↑	↔^c^	Alveolar and pulmonary vascular	Asthma (Habre et al., 2014); COPD (Schikowski et al., 2014); LRTIs (Asri et al., 2021); Lung cancer (Huang et al., 2017); ILD (Harari et al., 2020).
NO_2_	‐^a^	↓	↔	↔	Bronchioalveolar	Asthma (Guarnieri & Balmes, 2014); COPD (Doiron et al., 2019); ILD (Harari et al., 2020).
SO_2_	‐^a^	↑	↔	↑	Nasopharyngeal	Asthma (Bethel et al., 1984; Johns & Linn, 2011); reduced nasal function (Koenig et al., 1985).
Ozone	‐^a^	↔	↑↑	↑	Lower respiratory tract predominance	Asthma (Guarnieri & Balmes, 2014); ILD (Harari et al., 2020).

^a^ Gaseous pollutants; therefore, no particle size available.

^b^ The solubility of PM is high variable based on its composition.

^c^ The density of PM varies based on the composition of the material rather than its size.

Particle size, shape, and solubility are the predominant modulators of air pollutant deposition in the respiratory system (Weill, 2020). Particles of low density and low‐molar‐mass have greater diffusion coefficients and ability to form an airborne suspension through coalescing with other airborne material (Krzeszowiak et al., 2016). These particulates also have greater ability to deposit in distal airspaces due to their smaller size and greater Brownian motion, that is, random motion and frequent collisions with other particles (Xu et al., 2022). Highly soluble pollutants deposit in the upper respiratory tract through contact with airway surface liquid in mucous lined airways (Weill, 2020). Lower solubility pollutants have a greater tendency to deposit more distally, and because of the limited capabilities to cross the alveolar‐capillary membrane, tend to be retained in distal airspaces over weeks or months (Lippmann et al., 1980). Collectively, these factors modulate the respiratory symptoms experienced with inhalation of different mixtures of air pollutants.

### 3.2 Particulate matter

PM is the environmental air pollutant most associated with poor respiratory health outcomes (Fuller et al., 2022). PM refers to a suspension of solid or liquid matter in the air, often present within a mixture of other pollutants. PM is categorized into coarse (2.5–10 μm), fine (<2.5 μm), and ultrafine (<0.1 μm) sizes based on aerodynamic diameter. Most course particles (>2.5 μm) that are not filtered by nasal hairs are deposited in the mucus lined upper airways, although some course particles with low density may deposit more distally (Krzeszowiak et al., 2016). Fine particles (<2.5 μm) deposit more distally, with ultrafine particles entering the alveoli that do not have a protective mucus layer (Lippmann et al., 1980).

Air pollution (and PM) mainly propagates respiratory dysfunction through its action on the epithelium and immune cells such as macrophages, causing a pro‐inflammatory and pro‐oxidant state that damages various respiratory structures (Aghapour et al., 2022). The respiratory epithelium is not a passive structure and consists of numerous cell types that serve different functions; ciliated and mucous‐secreting cells supporting airway clearance, basal epithelial cells providing structural support and supporting repair of the epithelium cells through its progenitor function, and immune cells within the alveolar spaces which respond to inhaled foreign material (Misiukiewicz‐Stepien & Paplinska‐Goryca, 2021).

PM is primarily considered to have airway centric effects. PM induces epithelial barrier dysfunction in those with airways disease; some causing direct damage through formation of reactive oxygen species (ROS) in the epithelial lining fluid (Lakey et al., 2016) but most initiating damage through cellular pathways and alterations to mitochondrial function (Aghapour et al., 2022). Damage to the epithelial barrier propagates an exaggerated pro‐inflammatory responses and an upregulation of factors linked to airway remodeling (Iwanaga et al., 2013), key features of both uncontrolled asthma and COPD.

Several physiological mechanisms exist linking excess environmental air pollution exposure to the development of COPD, however a direct cause‐effect relationship has yet to be established (Schikowski et al., 2014). First, short‐ and long‐term exposure to excess PM have been linked to increased systemic inflammation markers such as C‐reactive protein, tumor necrosis factor (TNF)‐α, interleukin(IL)‐6 and IL‐8 (Li et al., 2022); all of which are typically elevated in those with COPD (Szalontai et al., 2021). Second, COPD is characterized by an imbalance between proteases and anti‐protease activity, leading to extracellular matrix degradation and airway remodeling (Szalontai et al., 2021). It is plausible, based on evidence from isolated studies (Ryu et al., 2022), that environmental PM exposure increases protease activity, leading to the degradation of elastin fibers and dysfunctional airway remodeling observed in COPD. However, further studies are needed to corroborate this hypothesis.

Metaplasia and hyperplasia of mucus‐secreting cells also occurs in in‐vivo models of PM exposure (He et al., 2017; Montgomery et al., 2020). Cilia function, which is responsible for 80%–90% of PM clearance in healthy, non‐smoking individuals (Krzeszowiak et al., 2016), is also reduced in response to PM exposure. This impairs airway clearance mechanisms and increases susceptibility to repeated cycles of inflammation and infection. In‐vivo models investigating the response of vagal afferent nerves to diesel tracheal infusions also suggest that increases vagal afferent nerve sensitivity may stimulate symptoms such as cough and bronchoconstriction (Robinson et al., 2018), showing the multifaceted downstream effects of PM exposures.

A key aspect that explains the diverse effects of PM exposure is its ability to coagulate with other environmental molecules (see Table 1). PM_2.5_ has a greater ability to be suspended in the air for long periods and readily binds to antigens and microorganisms (e.g., glycoproteins, bacterial polysaccharides, endotoxins, fungal spores, pollen, and enzymes) found in environmental air (Joubert et al., 2020). The oxidative potency of these combined molecules varies, as sources of contaminants such as metals (e.g., iron, copper, chromium, and zinc (Krzeszowiak et al., 2016; Mossman et al., 2007)) and allergens, vary with time and season. The effects of PM may therefore vary between allergic sensitisation (often via immunoglobulin E‐ (IgE) mechanisms (Joubert et al., 2020)) and irritant‐predominant effects, or both (Burge et al., 2012).

Evidence arising from a recent systematic review has identified an association between PM (and ozone and NO_2_) and the development of pulmonary fibrosis (Harari et al., 2020). Whilst the mechanisms of such an effect are unclear, several plausible mechanisms exist. First, distal retention of PM occurs more so for particle sizes less than 0.1 μm, with evidence of translocation to interstitial areas of the lungs between the conducting and alveolar regions (Berend, 2016). This may cause interstitial inflammation and fibrosis. Simulated in vitro PM exposures also show increased epithelial–mesenchymal transition, proliferation of alveolar type II epithelial cells and dysregulation of transforming growth factor‐β signaling (Johannson et al., 2015); key features linked to pulmonary fibrosis. The release of key inflammatory markers (e.g., IL‐4 and IL‐13) and ROS from macrophages and epithelial cells may also induce epigenetic changes linked to the progression of telomere shortening—a key finding associated with repetitive epithelial injury and aberrant wound healing in ILD (Wei et al., 2024).

### 3.3 Ozone

Ground‐level ozone is formed by photochemical reactions between sunlight and pollutant precursors, such as NOx and volatile organic compounds (Guarnieri & Balmes, 2014). Volatile organic compounds are chemicals containing at least one carbon and hydrogen atom, which readily aerosolise at room temperature (Rumchev et al., 2007). Ozone is a potent oxidizing agent which acts on lipids, proteins, fatty acids, and airway surface molecules lining the respiratory tract, causing epithelial barrier dysfunction (Schikowski et al., 2014). Environmental ozone levels are therefore associated with the exacerbation of numerous respiratory conditions (Duan et al., 2020; Harari et al., 2020; Shin et al., 2020).

Ozone has intermediate solubility, with irritant effects that effect the upper airway, bronchi, and surrounding parenchyma (Weill, 2020). At environmental levels (0.2–0.6 ppm), ozone has been associated with increased markers of cell damage in bronchioalveolar lavage samples (Aris et al., 1993), allergic sensitisation preceding new‐onset asthma in children (Kim et al., 2011) and increased airway hyperreactivity in response to direct bronchial challenge testing (Kehrl et al., 1999; Seltzer et al., 1986). Several studies have shown an increase in eosinophilic inflammation in response to ozone exposure (Aris et al., 1993; Seltzer et al., 1986), although these are primarily restricted to those with pre‐existing asthma, making it difficult to draw conclusions concerning causality.

Acute exposure to low levels of ozone (<70 ppb over 8‐h average) are known to be associated with declines in pulmonary function in children and the elderly (Holm & Balmes, 2022). The effect of ozone on pulmonary function in adults are less clear at concentrations from 1 to 42 ppb, with small magnitude effects on forced expiratory volume in one second (FEV_1_) and forced vital capacity (FVC) that are more prominent in the elderly and those exposed over multiple days (Holm & Balmes, 2022). Further research is required to understand the independent effects of ozone at environmental concentrations, accounting for common confounders such as smoking and pre‐existing asthma.

### 3.4 Oxidizing gases

Oxidizing gases, such as NO_2_ and SO_2_, are ubiquitous within environmental air and have concentration‐dependent actions on the respiratory system. NO_2_ is a low solubility oxidizing gas with a strong ability to penetrate into distal airspaces (Weill, 2020) and SO_2_ is highly soluble with effects that predominate in the upper airway. Similar to PM and ozone, the action of these oxidizing gases on the epithelium induces dysfunction through the generation of ROS and inflammatory markers, with well‐established associations to exacerbation of respiratory disease (Faustini et al., 2014; Koenig et al., 1985; Strand et al., 1997; Tunnicliffe et al., 1994).

High concentrations of NO_2_ cause a pattern of pneumonitis, which if left untreated, would lead to pulmonary fibrosis (Bauer et al., 1998). The impact of low level exposure to NO_2_ on the respiratory system are less prominent, with several studies suggesting its effects are negligible when co‐existing PM exposures are considered (Faustini et al., 2014; Robertson et al., 1984). NO_2_ levels are higher at sites of busy traffic, and several studies have shown changes in small airway function following exposure (Robinson et al., 2022; Schultz et al., 2016). Despite this, most adult studies show a relatively benign effect on spirometry (Kerr et al., 1979; Robertson et al., 1984). In children, however, even low‐level exposure to NO_2_ has been associated with pathology, with obstructive lung function often observed in those exposed to elevated NO_2_ levels (Moshammer et al., 2006).

The epithelial damage caused by NO_2_ instigates an immune response through the release of various cytokines and chemokines. Specifically, upregulation of IL‐4, IL‐5, and IL‐13, lead to increase eosinophilic airway inflammation (Bevelander et al., 2007), which is measurable via the assessment of fraction of exhaled nitric oxide (FeNO) (Chung, 2021). Co‐exposures of NO_2_ alongside PM_2.5_ have been shown to be associated with increased FeNO in pollution‐exposed school children (Zhang et al., 2021). Exposure of the epithelium to NO_2_ also increases the permeability and reactivity of the airways to allergens, with evidence showing increased sensitisation to house dust mite in the presence of increased domestic concentrations of NO_2_ in individuals with pre‐existing asthma (Tunnicliffe et al., 1994). During short‐term exposures at levels from 400 to 490 ppb, NO_2_ is also associated with increased bronchial hyper‐reactivity in response to allergen inhalation, supporting its role in sensitisation‐induced asthma (Devalia et al., 1994; Strand et al., 1997).

SO_2_ is a highly soluble pollutant that elicits bronchoconstriction in healthy subjects when inhaled orally (>5 ppb SO_2_), with more subtle reductions observed in peak expiratory flow (PEF) at concentrations >1 ppb (Johns & Linn, 2011). In those with asthma, the effects of environmental SO_2_ are enhanced, with increased adverse events requiring medical attention following acute exposure (Bethel et al., 1984; Koenig et al., 1985). In inner cities, mortality risk has been shown to increase with each 10 μg/m^3^ rise in SO_2_ (Orellano et al., 2017), suggesting an important role in the frequency and severity of exacerbation.

## 4 QUANTIFYING THE IMPACT OF AIR POLLUTION ON RESPIRATORY FUNCTION

The impact of air pollution on the respiratory system can be objectively quantified via the assessment of lung function and airway inflammation. Whilst there are several respiratory physiological tests available, spirometry and FeNO are most often used to evaluate the response to air pollution. Spirometric indices provide objective information in the assessment of dynamic lung volumes and airway caliber (Sylvester et al., 2020), although impulse oscillometry (a non‐volitional method of assessing respiratory mechanics) may be more sensitive to changes relating to pollution (Schultz et al., 2016).

In the general population, acute and chronic exposures to PM have been associated with reductions in FEV_1_ and FVC (Edginton et al., 2019). Specifically, each 10 μg/m^3^ increase in short‐term PM_2.5_ exposure and long‐term PM_10_ exposure has been shown to be associated with a 7–9 mL annual decline in annual FEV_1_. Whilst these findings appear trivial and in keeping with expected age‐related declines in lung function, it is important to consider that exposure levels to particulates may vary as much as 100 μg/m^3^ per week in developing countries (Hao et al., 2017), potentially contributing to respiratory decline in polluted areas.

The sensitivity of spirometry to detect early changes in respiratory function with exposure to air pollution is also sub‐optimal (Kim et al., 2020; Schultz et al., 2016). The reproducibility of the spirometry measurement and the duration of follow‐up are key parameters needed to assess the likelihood of significant lung function declines with air pollution over time (Hnizdo et al., 2005); each of which may vary and with different practice standards around the globe. The nature of the disease caused by air pollution also effects the sensitivity of conventional spirometry measures. In airways‐centric diseases, conventional spirometry measures may not detect changes in small airway function early in the disease process (Kim et al., 2020). In fibrotic conditions, spirometry may appear to be within a normal range and gas exchange abnormalities may manifest before volumetric changes occur within the lungs (Sylvester et al., 2021).

To date, not all studies have utilized the most appropriate measures to understand the respiratory impact of air pollution. Forced expiratory flow between 25% and 75% (FEF_25‐75_) has been used as a marker of small‐medium airway dysfunction in numerous studies (Berend, 2016). The use of FEF_25‐75_ is probematic, mainly due to the greater variation associated with the measurement and its reliance on maximal peak expiratory flow and FVC to generate reliable measurements.

## 5 SUSCEPTIBLE/HIGH RISK POPULATIONS

The exaggerated inflammatory response caused by air pollution inhalation is potentiated by suppression of antioxidant defenses (Liu et al., 2019). Individuals with reduced antioxidant defense pools or gene polymorphisms linked to reduced antioxidant activity (Chen et al., 2007) have greater levels of redox imbalance and lung function impairment in response to air pollution (Bowatte et al., 2017). Pathologies associated with hypersecretion and frequent cycles of bacterial colonization may therefore have a multi‐faceted inflammatory milieu contributing to disease (Aghapour et al., 2022; Dickerhof et al., 2017). This heightened state of inflammation propagated by long‐term exposure to air pollution has the potential to instigate and/or exacerbate chronic respiratory disease (Altman et al., 2023; Johannson et al., 2014).

### 5.1 Children

Children are at increased vulnerability to the effects of air pollution, due to the relatively higher respiratory rate and levels of ventilation per unit of body mass (Aithal et al., 2023). The greater nasal contribution to breathing in children, with less efficient nasal filtering in comparison to adults, increases the potential for large particles deposition in the upper airway (Bateson & Schwartz, 2007). Further, underdeveloped innate and adaptive immunity increases the potential for respiratory consequences from air pollution. Low level exposure to environmental air pollution is associated with allergic sensitisation and asthma onset in children (Gasana et al., 2012; Olsson et al., 2021). Respiratory diseases in adulthood often originate in childhood. Early adult lung function is also a strong predictor of respiratory health into the seventh decade of life (Bush, 2016). In longitudinal birth studies, PM exposure in childhood is associated with lower tidal volumes, higher respiratory rates and higher lung clearance index up to the first year of life (Gray et al., 2017; Lee et al., 2019), with increased peripheral airway resistance (Robinson et al., 2022; Schultz et al., 2016).

### 5.2 Elderly individuals

The cumulative exposure to environmental air pollution increases across the lifespan—alongside social, household, and occupational risk factors (Sandström et al., 2003). An age‐associated loss of lung function occurs after 30 years of age due to reduced lung elasticity and diaphragm strength (Agustí et al., 2017). In the distal airways, airflow obstruction and ventilation heterogeneity can occur as a consequence of reduced lung elasticity occurring with age, which increases particle deposition in peripheral airways (Segal et al., 2002). This is worsened by slowing of mucociliary clearance and cough; two factors essential to airway clearance of inhaled particles (Bailey, 2022). The antioxidant defense systems, which protect against oxidative pollutants, are also impaired with age (Liu et al., 2019), increasing susceptibility to exaggerated inflammatory responses and infection. As elderly individuals have lower levels of ventilatory reserve in absolute terms as well as co‐morbidities such as frailty, pollution‐related exacerbations have the potential to have life‐altering effects.

### 5.3 Pregnancy

The mother and baby have increased susceptibility to the effects of environmental air pollution from in‐utero exposures. In the first trimester of pregnancy, the mother experiences an increase in minute ventilation of up to 48% which is maintained throughout pregnancy in response to the increase in resting metabolism (LoMauro & Aliverti, 2015). The rise in minute ventilation is predominantly driven by an increase in resting tidal volumes, which reduces anatomical dead space and increases the likelihood of environmental air being deposited distally in the lungs. Lung development of the child in‐utero occurs up to 36‐weeks (third trimester) and inhaled particles sufficiently small to enter the circulation have the potential to interact with the developing foetus prior to full development (Bongaerts et al., 2022).

It is well‐known that noxious environmental air exposures, such as cigarette smoke (O'Shaughnessy et al., 2011), can alter foetal development, with further evidence that combustion‐derived particulates play a role in altering neonatal health outcomes (LoMauro & Aliverti, 2015). Adverse neonatal health outcomes including preterm birth (Mendola et al., 2016), low birth weight (Fleisch et al., 2015) and impaired lung function development (Latzin et al., 2009), have been reported in neonates in association with exposure to environmental air pollution. Impulse oscillometry has been particularly useful in terms of understanding the effects on the neonatal respiratory system, with evidence of increased small and large airway resistance in response to household and traffic‐related pollution exposures (Agyapong et al., 2023; Dutta et al., 2021). The effects of air pollution on the respiratory system can therefore manifest in‐utero and effect health outcomes throughout the life course.

### 5.4 Athletes

Elite and recreational endurance‐based athletes are exposed to significant environmental air pollution during training and competition. With progressive exercise, ventilation increases to match metabolic demand (Pritchard et al., 2021), initially via an increase in tidal volume, followed by an increase in breathing frequency at high workloads. In elite athletes, peak ventilation increases 20‐30‐fold higher at peak exercise (Price et al., 2019). A shift from nasal to combined oral and nasal airflow occurs above 30 L per minute, which exposes the distal airways to a higher volume of unfiltered and unconditioned air (e.g., exposure to cold dry air, pollutants, and/or aeroallergen) (Price, Walsted, et al., 2022). One in five athletes have evidence of lower airway dysfunction (i.e., >10% fall in FEV_1_ pre‐to‐post exercise) (Price, Sewry, et al., 2022). It is thought that regular participation in high‐intensity exercise in noxious environmental conditions may cause ‘airway injury’ promoting the development of airway dysfunction and respiratory symptoms (Kippelen & Anderson, 2012). On this basis, it has previously been argued that lower airway dysfunction in the context of competitive sport should be classified as an ‘occupational lung disease’—whereby elite athletes receive the same considerations for their respiratory health as others with relevant occupational exposures (Price et al., 2013).

### 5.5 Individuals with pre‐existing respiratory conditions

Air pollutants have the capacity to exacerbate pre‐existing respiratory disease through instigating epithelial barrier dyfunction and pro‐inflammatory and oxidant responses (Aghapour et al., 2022). Alterations to the respiratory tract may include inflammation, bronchoconstriction, and increased permeability and reactivity of the airways to irritants and allergens (Guarnieri & Balmes, 2014; Johannson et al., 2015). In those with impaired lung function and low ventilatory reserve, the risk of morbidity and mortality from air pollution‐mediated exacerbation is higher (Agustí et al., 2017), with epidemiological data demonstrating clear associations between environmental air pollution and healthcare utilization across a range of respiratory diseases (Perez et al., 2013). The incidence of respiratory disease follows a social gradient, with many individuals living in relative or absolute deprivation, where air pollution is often highest which confounds interpretation when considering individual effect (Marmot & Bell, 2018).

## 6 SUMMARY: CURRENT PERSPECTIVES AND FUTURE CHALLENGES

**FIGURE 1:**
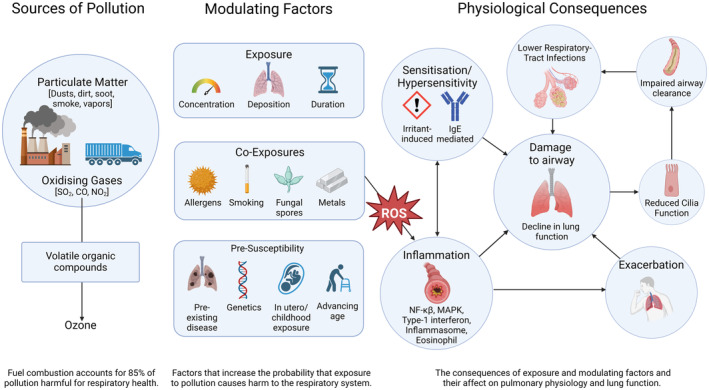
Physiological consequences of environmental air pollution on respiratory health and function.

Environmental air pollution presents a considerable risk to global respiratory health. As this review highlights, common airborne pollutants such as PM, ozone, and oxidizing gases stimulate respiratory inflammation, cilia dysfunction, airway remodeling, and respiratory symptoms such as cough. If critical levels are exceeded, inhaled pollutants can lead to the development of respiratory dysfunction and provoke exacerbation in those with pre‐existing chronic respiratory disease (Figure 1). Importantly, air pollution mitigation strategies are recognized to improve respiratory health outcomes (Gauderman et al., 2015), and therefore moving forward, a shared global effort is required to reduce the level of airborne contaminants, with policies reflecting disproportional effects according to vulnerable populations and geographical location.

In terms of evaluating the respiratory impact of environmental air pollution, traditional spirometry may lack the required diagnostic sensitivity to detect early signs of pathology. In several recent studies, airway resistance and reactance has been assessed using impulse oscillometry (Cottee et al., 2020; Kim et al., 2020; Postma et al., 2019)—thus identifying more subtle signs of respiratory disease (i.e., small airways dysfunction). Other advanced diagnostic technologies that have emerged in recent years (e.g., hyperpolarised magnetic resonance imaging and exhaled breath analytics (Stewart et al., 2022)) have been shown to provide utility in the assessment of respiratory symptoms where conventional pulmonary function tests remain inconclusive. A key focus for future research should therefore be to incorporate these emerging diagnostic techniques into epidemiolgical research to provide insight into pollutant‐specific exposure‐response relationships, with the ultimate aim of developing effective preventative strategies at both an individual and population‐based level.

## FUNDING INFORMATION

No funding was received.

## CONFLICT OF INTEREST STATEMENT

Dr. Oliver Price is an Associate Editor at Physiological Reports and was blinded from reviewing or making decisions for the manuscript. Another Editor oversaw the manuscript process for this article.

## References

[phy270006-bib-0001] Aghapour, M. , Ubags, N. D. , Bruder, D. , Hiemstra, P. S. , Sidhaye, V. , Rezaee, F. , & Heijink, I. H. (2022). Role of air pollutants in airway epithelial barrier dysfunction in asthma and COPD. European Respiratory Review, 31(163), 210112. 35321933 10.1183/16000617.0112-2021 PMC9128841

[phy270006-bib-0002] Agustí, A. , Noell, G. , Brugada, J. , & Faner, R. (2017). Lung function in early adulthood and health in later life: A transgenerational cohort analysis. The Lancet Respiratory Medicine, 5(12), 935–945. 29150410 10.1016/S2213-2600(17)30434-4

[phy270006-bib-0003] Agyapong, P. D. , Jack, D. , Kaali, S. , Colicino, E. , Mujtaba, M. N. , Chillrud, S. N. , Osei, M. , Gennings, C. , Agyei, O. , Kinney, P. L. , Kwarteng, A. , Perzanowski, M. , Dwommoh Prah, R. K. , Tawiah, T. , Asante, K. P. , & Lee, A. G. (2023). Household air pollution and child lung function: The Ghana randomized air pollution and health study. American Journal of Respiratory and Critical Care Medicine, 209, 716–726. 10.1164/rccm.202303-0623OC PMC12039047 38016085

[phy270006-bib-0004] Aithal, S. S. , Sachdeva, I. , & Kurmi, O. P. (2023). Air quality and respiratory health in children. Breathe, 19(2), 230040. 37377853 10.1183/20734735.0040-2023 PMC10292770

[phy270006-bib-0005] Altman, M. C. , Kattan, M. , O'Connor, G. T. , Murphy, R. C. , Whalen, E. , LeBeau, P. , Calatroni, A. , Gill, M. A. , Gruchalla, R. S. , Liu, A. H. , Lovinsky‐Desir, S. , Pongracic, J. A. , Kercsmar, C. M. , Khurana Hershey, G. K. , Zoratti, E. M. , Teach, S. J. , Bacharier, L. B. , Wheatley, L. M. , Sigelman, S. M. , … National Institute of Allergy and Infectious Disease's Inner City Asthma Consortium . (2023). Associations between outdoor air pollutants and non‐viral asthma exacerbations and airway inflammatory responses in children and adolescents living in urban areas in the USA: A retrospective secondary analysis. The Lancet Planetary Health, 7(1), e33–e44. 36608946 10.1016/S2542-5196(22)00302-3 PMC9984226

[phy270006-bib-0006] Aris, R. M. , Christian, D. , Hearne, P. Q. , Kerr, K. , Finkbeiner, W. E. , & Balmes, J. R. (1993). Ozone‐induced airway inflammation in human subjects as determined by airway lavage and biopsy. The American Review of Respiratory Disease, 148, 1363–1372. 8239177 10.1164/ajrccm/148.5.1363

[phy270006-bib-0007] Asri, A. K. , Pan, W.‐C. , Lee, H.‐Y. , Su, H.‐J. , Wu, C.‐D. , & Spengler, J. D. (2021). Spatial patterns of lower respiratory tract infections and their association with fine particulate matter. Scientific Reports, 11(1), 4866. 33649419 10.1038/s41598-021-84435-y PMC7921673

[phy270006-bib-0008] Baethge, C. , Goldbeck‐Wood, S. , & Mertens, S. (2019). SANRA—A scale for the quality assessment of narrative review articles. Research Integrity and Peer Review, 4, 1–7. 30962953 10.1186/s41073-019-0064-8 PMC6434870

[phy270006-bib-0009] Bailey, K. L. (2022). Aging diminishes Mucociliary clearance of the lung. Advances in Geriatric Medicine and Research, 4(2), 1–12. 10.20900/agmr20220005 PMC9435381 36066919

[phy270006-bib-0010] Bateson, T. F. , & Schwartz, J. (2007). Children's response to air pollutants. Journal of Toxicology and Environmental Health, Part A, 71(3), 238–243. 10.1080/15287390701598234 18097949

[phy270006-bib-0011] Bauer, U. , Berg, D. , Kohn, M. A. , Meriwether, R. A. , & Nickle, R. A. (1998). Acute effects of nitrogen dioxide after accidental release. Public Health Reports, 113(1), 62. 9475936 PMC1308370

[phy270006-bib-0012] Berend, N. (2016). Contribution of air pollution to COPD and small airway dysfunction. Respirology, 21(2), 237–244. 26412571 10.1111/resp.12644

[phy270006-bib-0013] Bethel, R. A. , Sheppard, D. , Epstein, J. , Tam, E. , Nadel, J. , & Boushey, H. A. (1984). Interaction of sulfur dioxide and dry cold air in causing bronchoconstriction in asthmatic subjects. Journal of Applied Physiology, 57(2), 419–423. 6469811 10.1152/jappl.1984.57.2.419

[phy270006-bib-0014] Bevelander, M. , Mayette, J. , Whittaker, L. A. , Paveglio, S. A. , Jones, C. C. , Robbins, J. , Hemenway, D. , Akira, S. , Uematsu, S. , & Poynter, M. E. (2007). Nitrogen dioxide promotes allergic sensitization to inhaled antigen. The Journal of Immunology, 179(6), 3680–3688. 17785804 10.4049/jimmunol.179.6.3680 PMC4697766

[phy270006-bib-0015] Bongaerts, E. , Lecante, L. L. , Bové, H. , Roeffaers, M. B. , Ameloot, M. , Fowler, P. A. , & Nawrot, T. S. (2022). Maternal exposure to ambient black carbon particles and their presence in maternal and fetal circulation and organs: An analysis of two independent population‐based observational studies. The Lancet Planetary Health, 6(10), e804–e811. 36208643 10.1016/S2542-5196(22)00200-5 PMC9553674

[phy270006-bib-0016] Bowatte, G. , Erbas, B. , Lodge, C. J. , Knibbs, L. D. , Gurrin, L. C. , Marks, G. B. , Thomas, P. S. , Johns, D. P. , Giles, G. G. , Hui, J. , Dennekamp, M. , Perret, J. L. , Abramson, M. J. , Walters, E. H. , Matheson, M. C. , & Dharmage, S. C. (2017). Traffic‐related air pollution exposure over a 5‐year period is associated with increased risk of asthma and poor lung function in middle age. The European Respiratory Journal, 50(4), 1602357. 29074540 10.1183/13993003.02357-2016

[phy270006-bib-0017] Burge, P. , Moore, V. , & Robertson, A. (2012). Sensitization and irritant‐induced occupational asthma with latency are clinically indistinguishable. Occupational Medicine, 62(2), 129–133. 22199365 10.1093/occmed/kqr211

[phy270006-bib-0018] Bush, A. (2016). Lung development and aging. Annals of the American Thoracic Society, 13(Supplement 5), S438–S446. 28005431 10.1513/AnnalsATS.201602-112AW

[phy270006-bib-0019] Chen, C. , Arjomandi, M. , Tager, I. B. , Holland, N. , & Balmes, J. R. (2007). Effects of antioxidant enzyme polymorphisms on ozone‐induced lung function changes. The European Respiratory Journal, 30(4), 677–683. 17652311 10.1183/09031936.00160806 PMC3282174

[phy270006-bib-0020] Chen, R. , Yin, P. , Meng, X. , Liu, C. , Wang, L. , Xu, X. , Ross, J. A. , Tse, L. A. , Zhao, Z. , Kan, H. , & Zhou, M. (2017). Fine particulate air pollution and daily mortality. A nationwide analysis in 272 Chinese cities. American Journal of Respiratory and Critical Care Medicine, 196(1), 73–81. 28248546 10.1164/rccm.201609-1862OC

[phy270006-bib-0021] Choi, J. , Sim, J. K. , Oh, J. Y. , Lee, Y. S. , Hur, G. Y. , Lee, S. Y. , Shim, J. J. , Moon, J. Y. , & Min, K. H. (2020). Relationship between particulate matter (PM10) and airway inflammation measured with exhaled nitric oxide test in Seoul, Korea. Canadian Respiratory Journal, 2020(1), 1823405. 32256904 10.1155/2020/1823405 PMC7103060

[phy270006-bib-0022] Chung, K. F. (2021). Increasing utility of FeNO as a biomarker of type‐2 inflammation in severe asthma. The Lancet Respiratory Medicine, 9(10), 1083–1084. 34181878 10.1016/S2213-2600(21)00170-3

[phy270006-bib-0023] Cohen, A. J. , Brauer, M. , Burnett, R. , Anderson, H. R. , Frostad, J. , Estep, K. , Balakrishnan, K. , Brunekreef, B. , Dandona, L. , Dandona, R. , Feigin, V. , Freedman, G. , Hubbell, B. , Jobling, A. , Kan, H. , Knibbs, L. , Liu, Y. , Martin, R. , Morawska, L. , … Forouzanfar, M. H. (2017). Estimates and 25‐year trends of the global burden of disease attributable to ambient air pollution: An analysis of data from the global burden of diseases study 2015. The Lancet, 389(10082), 1907–1918. 10.1016/S0140-6736(17)30505-6 PMC5439030 28408086

[phy270006-bib-0024] Cottee, A. M. , Seccombe, L. M. , Thamrin, C. , King, G. G. , Peters, M. J. , & Farah, C. S. (2020). Bronchodilator response assessed by the forced oscillation technique identifies poor asthma control with greater sensitivity than spirometry. Chest, 157(6), 1435–1441. 31982392 10.1016/j.chest.2019.12.035 PMC7268434

[phy270006-bib-0025] Devalia, J. , Rusznak, C. , Herdman, M. , Trigg, C. , Davies, R. , & Tarraf, H. (1994). Effect of nitrogen dioxide and sulphur dioxide on airway response of mild asthmatic patients to allergen inhalation. The Lancet, 344(8938), 1668–1671. 10.1016/s0140-6736(94)90458-8 7996960

[phy270006-bib-0026] Dickerhof, N. , Pearson, J. F. , Hoskin, T. S. , Berry, L. J. , Turner, R. , Sly, P. D. , Kettle, A. J. , & AREST CF . (2017). Oxidative stress in early cystic fibrosis lung disease is exacerbated by airway glutathione deficiency. Free Radical Biology & Medicine, 113, 236–243. 28982600 10.1016/j.freeradbiomed.2017.09.028

[phy270006-bib-0027] Doiron, D. , de Hoogh, K. , Probst‐Hensch, N. , Fortier, I. , Cai, Y. , De Matteis, S. , & Hansell, A. L. (2019). Air pollution, lung function and COPD: Results from the population‐based UK biobank study. The European Respiratory Journal, 54(1), 1802140. 31285306 10.1183/13993003.02140-2018

[phy270006-bib-0028] Duan, R.‐R. , Hao, K. , & Yang, T. (2020). Air pollution and chronic obstructive pulmonary disease. Chronic Diseases and Translational Medicine, 6(4), 260–269. 33336171 10.1016/j.cdtm.2020.05.004 PMC7729117

[phy270006-bib-0029] Dutta, A. , Alaka, M. , Ibigbami, T. , Adepoju, D. , Adekunle, S. , Olamijulo, J. , Adedokun, B. , Deji‐Abiodun, O. , Chartier, R. , Ojengbede, O. , & Olopade, C. O. (2021). Impact of prenatal and postnatal household air pollution exposure on lung function of 2‐year old Nigerian children by oscillometry. The Science of the Total Environment, 755, 143419. 33187696 10.1016/j.scitotenv.2020.143419

[phy270006-bib-0030] Edginton, S. , O'Sullivan, D. E. , King, W. , & Lougheed, M. D. (2019). Effect of outdoor particulate air pollution on FEV1 in healthy adults: A systematic review and meta‐analysis. Occupational and Environmental Medicine, 76(8), 583–591. 31189694 10.1136/oemed-2018-105420

[phy270006-bib-0031] Faustini, A. , Rapp, R. , & Forastiere, F. (2014). Nitrogen dioxide and mortality: Review and meta‐analysis of long‐term studies. The European Respiratory Journal, 44(3), 744–753. 24558178 10.1183/09031936.00114713

[phy270006-bib-0032] Fleisch, A. F. , Rifas‐Shiman, S. L. , Koutrakis, P. , Schwartz, J. D. , Kloog, I. , Melly, S. , Coull, B. A. , Zanobetti, A. , Gillman, M. W. , Gold, D. R. , & Oken, E. (2015). Prenatal exposure to traffic pollution: Associations with reduced fetal growth and rapid infant weight gain. Epidemiology (Cambridge, Mass.), 26(1), 43–50. 25437317 10.1097/EDE.0000000000000203 PMC4285344

[phy270006-bib-0033] Fuller, R. , Landrigan, P. J. , Balakrishnan, K. , Bathan, G. , Bose‐O'Reilly, S. , Brauer, M. , Caravanos, J. , Chiles, T. , Cohen, A. , Corra, L. , Cropper, M. , Ferraro, G. , Hanna, J. , Hanrahan, D. , Hu, H. , Hunter, D. , Janata, G. , Kupka, R. , Lanphear, B. , … Yan, C. (2022). Pollution and health: A progress update. The Lancet Planetary Health, 6(6), e535–e547.35594895 10.1016/S2542-5196(22)00090-0

[phy270006-bib-0034] Gasana, J. , Dillikar, D. , Mendy, A. , Forno, E. , & Vieira, E. R. (2012). Motor vehicle air pollution and asthma in children: A meta‐analysis. Environmental Research, 117, 36–45. 22683007 10.1016/j.envres.2012.05.001

[phy270006-bib-0035] Gauderman, W. J. , Urman, R. , Avol, E. , Berhane, K. , McConnell, R. , Rappaport, E. , Chang, R. , Lurmann, F. , & Gilliland, F. (2015). Association of improved air quality with lung development in children. The New England Journal of Medicine, 372(10), 905–913. 25738666 10.1056/NEJMoa1414123 PMC4430551

[phy270006-bib-0036] Gourd, E. (2022). New evidence that air pollution contributes substantially to lung cancer. The Lancet Oncology, 23(10), e448. 36116453 10.1016/S1470-2045(22)00569-1

[phy270006-bib-0037] Gray, D. M. , Turkovic, L. , Willemse, L. , Visagie, A. , Vanker, A. , Stein, D. J. , Sly, P. D. , Hall, G. L. , & Zar, H. J. (2017). Lung function in African infants in the Drakenstein child health study. Impact of lower respiratory tract illness. American Journal of Respiratory and Critical Care Medicine, 195(2), 212–220. 27509359 10.1164/rccm.201601-0188OC PMC5394784

[phy270006-bib-0038] Guarnieri, M. , & Balmes, J. R. (2014). Outdoor air pollution and asthma. The Lancet, 383(9928), 1581–1592. 10.1016/S0140-6736(14)60617-6 PMC4465283 24792855

[phy270006-bib-0039] Guo, C. , Zhang, Z. , Lau, A. K. , Lin, C. Q. , Chuang, Y. C. , Chan, J. , Jiang, W. K. , Tam, T. , Yeoh, E. K. , Chan, T. C. , Chang, L. Y. , & Lao, X. Q. (2018). Effect of long‐term exposure to fine particulate matter on lung function decline and risk of chronic obstructive pulmonary disease in Taiwan: A longitudinal, cohort study. The Lancet Planetary Health, 2(3), e114–e125. 29615226 10.1016/S2542-5196(18)30028-7

[phy270006-bib-0040] Habre, R. , Moshier, E. , Castro, W. , Nath, A. , Grunin, A. , Rohr, A. , Godbold, J. , Schachter, N. , Kattan, M. , Coull, B. , & Koutrakis, P. (2014). The effects of PM2. 5 and its components from indoor and outdoor sources on cough and wheeze symptoms in asthmatic children. Journal of Exposure Science & Environmental Epidemiology, 24(4), 380–387. 24714073 10.1038/jes.2014.21

[phy270006-bib-0041] Hao, Y. , Zhang, G. , Han, B. , Xu, X. , Feng, N. , Li, Y. , Wang, W. , Kan, H. , Bai, Z. , Zhu, Y. , Au, W. , & Xia, Z. L. (2017). Prospective evaluation of respiratory health benefits from reduced exposure to airborne particulate matter. International Journal of Environmental Health Research, 27(2), 126–135. 28245677 10.1080/09603123.2017.1292497

[phy270006-bib-0042] Harari, S. , Raghu, G. , Caminati, A. , Cruciani, M. , Franchini, M. , & Mannucci, P. (2020). Fibrotic interstitial lung diseases and air pollution: A systematic literature review. European Respiratory Review, 29(157), 200093. 32817115 10.1183/16000617.0093-2020 PMC9488644

[phy270006-bib-0043] He, F. , Liao, B. , Pu, J. , Li, C. , Zheng, M. , Huang, L. , Zhou, Y. , Zhao, D. , Li, B. , & Ran, P. (2017). Exposure to ambient particulate matter induced COPD in a rat model and a description of the underlying mechanism. Scientific Reports, 7(1), 45666. 28361885 10.1038/srep45666 PMC5374504

[phy270006-bib-0044] Hnizdo, E. , Yu, L. , Freyder, L. , Attfield, M. , Lefante, J. , & Glindmeyer, H. (2005). The precision of longitudinal lung function measurements: Monitoring and interpretation. Occupational and Environmental Medicine, 62(10), 695–701. 16169915 10.1136/oem.2004.018424 PMC1740862

[phy270006-bib-0045] Holm, S. M. , & Balmes, J. R. (2022). Systematic review of ozone effects on human lung function, 2013 through 2020. Chest, 161(1), 190–201. 34389296 10.1016/j.chest.2021.07.2170 PMC8783034

[phy270006-bib-0046] Huang, F. , Pan, B. , Wu, J. , Chen, E. , & Chen, L. (2017). Relationship between exposure to PM2. 5 and lung cancer incidence and mortality: A meta‐analysis. Oncotarget, 8(26), 43322. 28487493 10.18632/oncotarget.17313 PMC5522148

[phy270006-bib-0047] Hunt, A. , Abraham, J. L. , Judson, B. , & Berry, C. L. (2003). Toxicologic and epidemiologic clues from the characterization of the 1952 London smog fine particulate matter in archival autopsy lung tissues. Environmental Health Perspectives, 111(9), 1209–1214. 12842775 10.1289/ehp.6114 PMC1241576

[phy270006-bib-0048] Iwanaga, K. , Elliott, M. S. , Vedal, S. , & Debley, J. S. (2013). Urban particulate matter induces pro‐remodeling factors by airway epithelial cells from healthy and asthmatic children. Inhalation Toxicology, 25(12), 653–660. 24102466 10.3109/08958378.2013.827283

[phy270006-bib-0049] Johannson, K. A. , Balmes, J. R. , & Collard, H. R. (2015). Air pollution exposure: A novel environmental risk factor for interstitial lung disease? Chest, 147(4), 1161–1167. 25846532 10.1378/chest.14-1299 PMC4388120

[phy270006-bib-0050] Johannson, K. A. , Vittinghoff, E. , Lee, K. , Balmes, J. R. , Ji, W. , Kaplan, G. G. , Kim, D. S. , & Collard, H. R. (2014). Acute exacerbation of idiopathic pulmonary fibrosis associated with air pollution exposure. The European Respiratory Journal, 43(4), 1124–1131. 24176998 10.1183/09031936.00122213 PMC5555605

[phy270006-bib-0051] Johns, D. O. , & Linn, W. S. (2011). A review of controlled human SO2 exposure studies contributing to the US EPA integrated science assessment for sulfur oxides. Inhalation Toxicology, 23(1), 33–43. 21222560 10.3109/08958378.2010.539290

[phy270006-bib-0052] Joubert, A. I. , Geppert, M. , Johnson, L. , Mills‐Goodlet, R. , Michelini, S. , Korotchenko, E. , Duschl, A. , Weiss, R. , Horejs‐Höck, J. , & Himly, M. (2020). Mechanisms of particles in sensitization, effector function and therapy of allergic disease. Frontiers in Immunology, 11, 1334. 32714326 10.3389/fimmu.2020.01334 PMC7344151

[phy270006-bib-0053] Kehrl, H. R. , Peden, D. B. , Ball, B. , Folinsbee, L. J. , & Horstman, D. (1999). Increased specific airway reactivity of persons with mild allergic asthma after 7.6 hours of exposure to 0.16 ppm ozone. The Journal of Allergy and Clinical Immunology, 104(6), 1198–1204. 10589001 10.1016/s0091-6749(99)70013-8

[phy270006-bib-0054] Kerr, H. D. , Kulle, T. J. , McIlhany, M. L. , & Swidersky, P. (1979). Effects of nitrogen dioxide on pulmonary function in human subjects: An environmental chamber study. Environmental Research, 19(2), 392–404. 499157 10.1016/0013-9351(79)90064-1

[phy270006-bib-0055] Keswani, A. , Akselrod, H. , & Anenberg, S. C. (2022). Health and clinical impacts of air pollution and linkages with climate change. NEJM Evidence, 1(7), EVIDra2200068. 38319260 10.1056/EVIDra2200068

[phy270006-bib-0056] Kim, B.‐J. , Kwon, J.‐W. , Seo, J.‐H. , Kim, H.‐B. , Lee, S.‐Y. , Park, K.‐S. , Yu, J. , Kim, H. C. , Leem, J. H. , Sakong, J. , Kim, S. Y. , Lee, C. G. , Kang, D. M. , Ha, M. , Hong, Y. C. , Kwon, H. J. , & Hong, S. J. (2011). Association of ozone exposure with asthma, allergic rhinitis, and allergic sensitization. Annals of Allergy, Asthma & Immunology, 107(3), 214–219. e1. 10.1016/j.anai.2011.05.025 21875539

[phy270006-bib-0057] Kim, S.‐R. , Park, K. H. , Son, N.‐H. , Moon, J. , Park, H. J. , Kim, K. , Park, J. W. , & Lee, J. H. (2020). Application of impulse oscillometry in adult asthmatic patients with preserved lung function. Allergy, Asthma & Immunology Research, 12(5), 832–843. 10.4168/aair.2020.12.5.832 PMC7346993 32638563

[phy270006-bib-0058] Kippelen, P. , & Anderson, S. D. (2012). Airway injury during high‐level exercise. British Journal of Sports Medicine, 46(6), 385–390. 22247295 10.1136/bjsports-2011-090819

[phy270006-bib-0059] Koenig, J. Q. , Morgan, M. S. , Horike, M. , & Pierson, W. E. (1985). The effects of sulfur oxides on nasal and lung function in adolescents with extrinsic asthma. The Journal of Allergy and Clinical Immunology, 76(6), 813–818. 4067130 10.1016/0091-6749(85)90754-7

[phy270006-bib-0060] Krzeszowiak, J. , Stefanow, D. , & Pawlas, K. (2016). The impact of particulate matter (PM) and nitric oxides (NOx) on human health and an analysis of selected sources accounting for their emission in Poland. Medycyna Środowiskowa‐Environmental Medicine, 19(3), 7–15.

[phy270006-bib-0061] Lakey, P. S. , Berkemeier, T. , Tong, H. , Arangio, A. M. , Lucas, K. , Pöschl, U. , & Shiraiwa, M. (2016). Chemical exposure‐response relationship between air pollutants and reactive oxygen species in the human respiratory tract. Scientific Reports, 6(1), 32916. 27605301 10.1038/srep32916 PMC5015057

[phy270006-bib-0062] Landrigan, P. J. (2017). Air pollution and health. The Lancet Public Health, 2(1), e4–e5. 29249479 10.1016/S2468-2667(16)30023-8

[phy270006-bib-0063] Latzin, P. , Röösli, M. , Huss, A. , Kuehni, C. E. , & Frey, U. (2009). Air pollution during pregnancy and lung function in newborns: A birth cohort study. The European Respiratory Journal, 33(3), 594–603. 19010988 10.1183/09031936.00084008

[phy270006-bib-0064] Lee, A. G. , Kaali, S. , Quinn, A. , Delimini, R. , Burkart, K. , Opoku‐Mensah, J. , Wylie, B. J. , Yawson, A. K. , Kinney, P. L. , Ae‐Ngibise, K. A. , Chillrud, S. , Jack, D. , & Asante, K. P. (2019). Prenatal household air pollution is associated with impaired infant lung function with sex‐specific effects. Evidence from GRAPHS, a cluster randomized cookstove intervention trial. American Journal of Respiratory and Critical Care Medicine, 199(6), 738–746. 30256656 10.1164/rccm.201804-0694OC PMC6423100

[phy270006-bib-0065] Lelieveld, J. , Evans, J. S. , Fnais, M. , Giannadaki, D. , & Pozzer, A. (2015). The contribution of outdoor air pollution sources to premature mortality on a global scale. Nature, 525(7569), 367–371. 26381985 10.1038/nature15371

[phy270006-bib-0066] Li, T. , Yu, Y. , Sun, Z. , & Duan, J. (2022). A comprehensive understanding of ambient particulate matter and its components on the adverse health effects based from epidemiological and laboratory evidence. Particle and Fibre Toxicology, 19(1), 67. 36447278 10.1186/s12989-022-00507-5 PMC9707232

[phy270006-bib-0067] Li, X. , Jin, L. , & Kan, H. (2019). Air pollution: A global problem needs local fixes. Nature, 570(7762), 437–439. 31239571 10.1038/d41586-019-01960-7

[phy270006-bib-0068] Lin, L. , Li, T. , Sun, M. , Liang, Q. , Ma, Y. , Wang, F. , Duan, J. , & Sun, Z. (2021). Effect of particulate matter exposure on the prevalence of allergic rhinitis in children: A systematic review and meta‐analysis. Chemosphere, 268, 128841. 33172665 10.1016/j.chemosphere.2020.128841

[phy270006-bib-0069] Lippmann, M. , Yeates, D. , & Albert, R. (1980). Deposition, retention, and clearance of inhaled particles. Occupational and Environmental Medicine, 37(4), 337–362. 10.1136/oem.37.4.337 PMC1008751 7004477

[phy270006-bib-0070] Liu, X. , Wang, J. , Fan, Y. , Xu, Y. , Xie, M. , Yuan, Y. , Li, H. , & Qian, X. (2019). Particulate matter exposure history affects antioxidant defense response of mouse lung to haze episodes. Environmental Science & Technology, 53(16), 9789–9799. 31328514 10.1021/acs.est.9b01068

[phy270006-bib-0071] LoMauro, A. , & Aliverti, A. (2015). Respiratory physiology of pregnancy: Physiology masterclass. Breathe, 11(4), 297–301. 27066123 10.1183/20734735.008615 PMC4818213

[phy270006-bib-0072] Marmot, M. , & Bell, R. (2018). The sustainable development goals and health equity. Epidemiology, 29(1), 5–7. 29053554 10.1097/EDE.0000000000000773

[phy270006-bib-0073] Mendola, P. , Wallace, M. , Hwang, B. S. , Liu, D. , Robledo, C. , Männistö, T. , Sundaram, R. , Sherman, S. , Ying, Q. , & Grantz, K. L. (2016). Preterm birth and air pollution: Critical windows of exposure for women with asthma. The Journal of Allergy and Clinical Immunology, 138(2), 432–440. e5. 26944405 10.1016/j.jaci.2015.12.1309 PMC4975980

[phy270006-bib-0074] Misiukiewicz‐Stepien, P. , & Paplinska‐Goryca, M. (2021). Biological effect of PM10 on airway epithelium‐focus on obstructive lung diseases. Clinical Immunology, 227, 108754. 33964432 10.1016/j.clim.2021.108754

[phy270006-bib-0075] Montgomery, M. T. , Sajuthi, S. P. , Cho, S.‐H. , Everman, J. L. , Rios, C. L. , Goldfarbmuren, K. C. , Jackson, N. D. , Saef, B. , Cromie, M. , Eng, C. , Medina, V. , Elhawary, J. R. , Oh, S. S. , Rodriguez‐Santana, J. , Vladar, E. K. , Burchard, E. G. , & Seibold, M. A. (2020). Genome‐wide analysis reveals mucociliary remodeling of the nasal airway epithelium induced by urban PM2. 5. American Journal of Respiratory Cell and Molecular Biology, 63(2), 172–184. 32275839 10.1165/rcmb.2019-0454OC PMC7397762

[phy270006-bib-0076] Moser, C. , & Satterthwaite, D. (2010). Toward pro‐poor adaptation to climate change in the urban centers of low‐and middle‐income countries (pp. 231–258). Equity and vulnerability in a warming world.

[phy270006-bib-0077] Moshammer, H. , Hutter, H. , Hauck, H. , & Neuberger, M. (2006). Low levels of air pollution induce changes of lung function in a panel of schoolchildren. The European Respiratory Journal, 27(6), 1138–1143. 16455832 10.1183/09031936.06.00089605

[phy270006-bib-0078] Mossman, B. T. , Borm, P. J. , Castranova, V. , Costa, D. L. , Donaldson, K. , & Kleeberger, S. R. (2007). Mechanisms of action of inhaled fibers, particles and nanoparticles in lung and cardiovascular diseases. Particle and Fibre Toxicology, 4(1), 1–10. 17537262 10.1186/1743-8977-4-4 PMC1894816

[phy270006-bib-0079] OECD I . Energy and air pollution: World energy outlook special report 2016. 2016. International Energy Agency

[phy270006-bib-0080] Olsson, D. , Forsberg, B. , Bråbäck, L. , Geels, C. , Brandt, J. , Christensen, J. H. , Frohn, L. M. , & Oudin, A. (2021). Early childhood exposure to ambient air pollution is associated with increased risk of paediatric asthma: An administrative cohort study from Stockholm, Sweden. Environment International, 155, 106667. 34077855 10.1016/j.envint.2021.106667

[phy270006-bib-0081] Orellano, P. , Quaranta, N. , Reynoso, J. , Balbi, B. , & Vasquez, J. (2017). Effect of outdoor air pollution on asthma exacerbations in children and adults: Systematic review and multilevel meta‐analysis. PLoS One, 12(3), e0174050. 28319180 10.1371/journal.pone.0174050 PMC5358780

[phy270006-bib-0082] O'Shaughnessy, P. J. , Monteiro, A. , Bhattacharya, S. , & Fowler, P. A. (2011). Maternal smoking and fetal sex significantly affect metabolic enzyme expression in the human fetal liver. The Journal of Clinical Endocrinology & Metabolism, 96(9), 2851–2860. 21715529 10.1210/jc.2011-1437

[phy270006-bib-0083] Perez, L. , Declercq, C. , Iñiguez, C. , Aguilera, I. , Badaloni, C. , Ballester, F. , Bouland, C. , Chanel, O. , Cirarda, F. B. , Forastiere, F. , Forsberg, B. , Haluza, D. , Hedlund, B. , Cambra, K. , Lacasaña, M. , Moshammer, H. , Otorepec, P. , Rodríguez‐Barranco, M. , Medina, S. , & Künzli, N. (2013). Chronic burden of near‐roadway traffic pollution in 10 European cities (APHEKOM network). The European Respiratory Journal, 42(3), 594–605. 23520318 10.1183/09031936.00031112

[phy270006-bib-0084] Postma, D. S. , Brightling, C. , Baldi, S. , Van den Berge, M. , Fabbri, L. M. , Gagnatelli, A. , Papi, A. , Van der Molen, T. , Rabe, K. F. , Siddiqui, S. , Singh, D. , Nicolini, G. , Kraft, M. , & ATLANTIS study group . (2019). Exploring the relevance and extent of small airways dysfunction in asthma (ATLANTIS): Baseline data from a prospective cohort study. The Lancet Respiratory Medicine, 7(5), 402–416. 30876830 10.1016/S2213-2600(19)30049-9

[phy270006-bib-0085] Price, O. , Walsted, E. , & Hull, J. (2019). Understanding the total airway response to exercise: Current perspectives and future challenges. Current Opinion in Physiology, 10, 185–192.

[phy270006-bib-0086] Price, O. J. , Ansley, L. , Menzies‐Gow, A. , Cullinan, P. , & Hull, J. H. (2013). Airway dysfunction in elite athletes–an occupational lung disease? Allergy, 68(11), 1343–1352. 24117544 10.1111/all.12265

[phy270006-bib-0087] Price, O. J. , Sewry, N. , Schwellnus, M. , Backer, V. , Reier‐Nilsen, T. , Bougault, V. , Pedersen, L. , Chenuel, B. , Larsson, K. , & Hull, J. H. (2022). Prevalence of lower airway dysfunction in athletes: A systematic review and meta‐analysis by a subgroup of the IOC consensus group on ‘acute respiratory illness in the athlete’. British Journal of Sports Medicine, 56(4), 213–222. 34872908 10.1136/bjsports-2021-104601

[phy270006-bib-0088] Price, O. J. , Walsted, E. S. , Bonini, M. , Brannan, J. D. , Bougault, V. , Carlsen, K. H. , Couto, M. , Kippelen, P. , Moreira, A. , Pite, H. , Rukhadze, M. , & Hull, J. H. (2022). Diagnosis and management of allergy and respiratory disorders in sport: An EAACI task force position paper. Allergy, 77(10), 2909–2923. 35809082 10.1111/all.15431 PMC9796481

[phy270006-bib-0089] Pritchard, A. , Burns, P. , Correia, J. , Jamieson, P. , Moxon, P. , Purvis, J. , Thomas, M. , Tighe, H. , & Sylvester, K. P. (2021). ARTP statement on cardiopulmonary exercise testing 2021. BMJ Open Respiratory Research, 8(1), e001121. 10.1136/bmjresp-2021-001121 PMC8593741 34782330

[phy270006-bib-0090] Robertson, A. , Dodgson, J. , Collings, P. , & Seaton, A. (1984). Exposure to oxides of nitrogen: Respiratory symptoms and lung function in British coalminers. Occupational and Environmental Medicine, 41(2), 214–219. 10.1136/oem.41.2.214 PMC1009286 6722049

[phy270006-bib-0091] Robinson, P. D. , Salimi, F. , Cowie, C. T. , Clifford, S. , King, G. G. , Thamrin, C. , Hardaker, K. , Mazaheri, M. , Morawska, L. , Toelle, B. G. , & Marks, G. B. (2022). Ultrafine particle exposure and biomarkers of effect on small airways in children. Environmental Research, 214, 113860. 35820650 10.1016/j.envres.2022.113860

[phy270006-bib-0092] Robinson, R. K. , Birrell, M. A. , Adcock, J. J. , Wortley, M. A. , Dubuis, E. D. , Chen, S. , McGilvery, C. M. , Hu, S. , Shaffer, M. S. P. , Bonvini, S. J. , Maher, S. A. , Mudway, I. S. , Porter, A. E. , Carlsten, C. , Tetley, T. D. , & Belvisi, M. G. (2018). Mechanistic link between diesel exhaust particles and respiratory reflexes. The Journal of Allergy and Clinical Immunology, 141(3), 1074–1084. 28532657 10.1016/j.jaci.2017.04.038 PMC5840514

[phy270006-bib-0093] Rumchev, K. , Brown, H. , & Spickett, J. (2007). Volatile organic compounds: Do they present a risk to our health? Reviews on Environmental Health, 22(1), 39–56. 17508697 10.1515/reveh.2007.22.1.39

[phy270006-bib-0094] Ryu, M. H. , Afshar, T. , Li, H. , Wooding, D. J. , Orach, J. , Zhou, J. S. , Murphy, S. , Lau, K. S. K. , Schwartz, C. , Yuen, A. C. Y. , Rider, C. F. , & Carlsten, C. (2022). Impact of exposure to diesel exhaust on inflammation markers and proteases in former smokers with chronic obstructive pulmonary disease: A randomized, double‐blinded, crossover study. American Journal of Respiratory and Critical Care Medicine, 205(9), 1046–1052. 35202552 10.1164/rccm.202104-1079OC

[phy270006-bib-0095] Sandström, T. , Frew, A. , Svartengren, M. , & Viegi, G. (2003). The need for a focus on air pollution research in the elderly. The European Respiratory Journal, 21(40 suppl), 92s–95s. 10.1183/09031936.03.00403503 12762582

[phy270006-bib-0096] Schikowski, T. , Mills, I. C. , Anderson, H. R. , Cohen, A. , Hansell, A. , Kauffmann, F. , Kramer, U. , Marcon, A. , Perez, L. , Sunyer, J. , Probst‐Hensch, N. , & Kunzli, N. (2014). Ambient air pollution: A cause of COPD? The European Respiratory Journal, 43(1), 250–263. 23471349 10.1183/09031936.00100112

[phy270006-bib-0097] Schultz, E. S. , Hallberg, J. , Gustafsson, P. M. , Bottai, M. , Bellander, T. , Bergström, A. , Kull, I. , Gruzieva, O. , Thunqvist, P. , Pershagen, G. , & Melén, E. (2016). Early life exposure to traffic‐related air pollution and lung function in adolescence assessed with impulse oscillometry. The Journal of Allergy and Clinical Immunology, 138(3), 930–932. 27297996 10.1016/j.jaci.2016.04.014

[phy270006-bib-0098] Segal, R. , Martonen, T. , Kim, C. , & Shearer, M. (2002). Computer simulations of particle deposition in the lungs of chronic obstructive pulmonary disease patients. Inhalation Toxicology, 14(7), 705–720. 12122571 10.1080/08958370290084593

[phy270006-bib-0099] Seltzer, J. , Bigby, B. G. , Stulbarg, M. , Holtzman, M. J. , Nadel, J. A. , Ueki, I. F. , Leikauf, G. D. , Goetzl, E. J. , & Boushey, H. A. (1986). O3‐induced change in bronchial reactivity to methacholine and airway inflammation in humans. Journal of Applied Physiology, 60(4), 1321–1326. 3084448 10.1152/jappl.1986.60.4.1321

[phy270006-bib-0100] Shin, S.‐W. , Bae, D.‐J. , Park, C.‐S. , Lee, J.‐U. , Kim, R.‐H. , Kim, S. R. , Chang, H. S. , & Park, J. S. (2020). Effects of air pollution on moderate and severe asthma exacerbations. The Journal of Asthma, 57(8), 875–885. 31122089 10.1080/02770903.2019.1611844

[phy270006-bib-0101] Stewart, N. J. , Smith, L. J. , Chan, H.‐F. , Eaden, J. A. , Rajaram, S. , Swift, A. J. , Weatherley, N. D. , Biancardi, A. , Collier, G. J. , Hughes, D. , Klafkowski, G. , Johns, C. S. , West, N. , Ugonna, K. , Bianchi, S. M. , Lawson, R. , Sabroe, I. , Marshall, H. , & Wild, J. M. (2022). Lung MRI with hyperpolarised gases: Current & future clinical perspectives. The British Journal of Radiology, 95(1132), 20210207. 34106792 10.1259/bjr.20210207 PMC9153706

[phy270006-bib-0102] Strand, V. , Rak, S. , Svartengren, M. , & Bylin, G. (1997). Nitrogen dioxide exposure enhances asthmatic reaction to inhaled allergen in subjects with asthma. American Journal of Respiratory and Critical Care Medicine, 155(3), 881–887. 9117021 10.1164/ajrccm.155.3.9117021

[phy270006-bib-0103] Sylvester, K. P. , Clayton, N. , Cliff, I. , Hepple, M. , Kendrick, A. , Kirkby, J. , Miller, M. , Moore, A. , Rafferty, G. F. , O'Reilly, L. , Shakespeare, J. , Smith, L. , Watts, T. , Bucknall, M. , & Butterfield, K. (2020). ARTP statement on pulmonary function testing 2020. BMJ open. Respiratory Research, 7(1), e000575. 10.1136/bmjresp-2020-000575 PMC7337892 32631927

[phy270006-bib-0104] Sylvester, K. P. , Youngs, L. , Rutter, M. , Beech, R. , & Mahadeva, R. (2021). Early respiratory diagnosis: Benefits of enhanced lung function assessment. BMJ Open Respiratory Research, 8(1), e001012. 10.1136/bmjresp-2021-001012 PMC8317074 34312255

[phy270006-bib-0105] Szalontai, K. , Gémes, N. , Furák, J. , Varga, T. , Neuperger, P. , Balog, J. Á. , Puskás, L. G. , & Szebeni, G. J. (2021). Chronic obstructive pulmonary disease: Epidemiology, biomarkers, and paving the way to lung cancer. Journal of Clinical Medicine, 10(13), 2889. 34209651 10.3390/jcm10132889 PMC8268950

[phy270006-bib-0106] Tunnicliffe, W. , Burge, P. , & Ayres, J. (1994). Effect of domestic concentrations of nitrogen dioxide on airway responses to inhaled allergen in asthmatic patients. The Lancet, 344(8939–8940), 1733–1736. 10.1016/s0140-6736(94)92886-x 7997002

[phy270006-bib-0107] Wei, B. , Zhou, Y. , Li, Q. , Zhen, S. , Wu, Q. , Xiao, Z. , Liao, J. , Zhu, B. , Duan, J. , Yang, X. , & Liang, F. (2024). Outdoor fine particulate matter exposure and telomere length in humans: A systematic review and meta‐analysis. Ecotoxicology and Environmental Safety, 275, 116206. 38518608 10.1016/j.ecoenv.2024.116206

[phy270006-bib-0108] Weill H. Occupational lung diseases: Research approaches and methods. 2020. Routledge

[phy270006-bib-0109] WHO . Ambient (outdoor) air pollution: Key facts 2022. https://www.who.int/news‐room/fact‐sheets/detail/ambient‐(outdoor)‐air‐quality‐and‐health.

[phy270006-bib-0110] Xu, Z. , Li, J. , & Han, Z. (2022). Numerical study of particle fouling deposition on heat transfer surface. Energy Storage and Saving, 1(1), 44–52.

[phy270006-bib-0111] Zemp, E. , Elsasser, S. , Schindler, C. , Kunzli, N. , Perruchoud, A. P. , Domenighetti, G. , Medici, T. , Ackermann‐Liebrich, U. , Leuenberger, P. , Monn, C. , Bolognini, G. , Bongard, J. P. , Brändli, O. , Karrer, W. , Keller, R. , Schöni, M. H. , Tschopp, J. M. , Villiger, B. , & Zellweger, J. P. (1999). Long‐term ambient air pollution and respiratory symptoms in adults (SAPALDIA study). American Journal of Respiratory and Critical Care Medicine, 159(4), 1257–1266. 10194174 10.1164/ajrccm.159.4.9807052

[phy270006-bib-0112] Zhang, Y. , Eckel, S. P. , Berhane, K. , Garcia, E. , Muchmore, P. , Molshatzki, N. B.‐A. , Rappaport, E. B. , Linn, W. S. , Habre, R. , & Gilliland, F. D. (2021). Long‐term exposures to air pollutants affect FeNO in children: A longitudinal study. The European Respiratory Journal, 58(5), 2100705. 34503981 10.1183/13993003.00705-2021 PMC9618404

[phy270006-bib-0113] Ziou, M. , Tham, R. , Wheeler, A. J. , Zosky, G. R. , Stephens, N. , & Johnston, F. H. (2022). Outdoor particulate matter exposure and upper respiratory tract infections in children and adolescents: A systematic review and meta‐analysis. Environmental Research, 210, 112969. 35183515 10.1016/j.envres.2022.112969

